# Job Satisfaction Analysis in Rural China: A Qualitative Study of Doctors in a Township Hospital

**DOI:** 10.1155/2017/1964087

**Published:** 2017-03-19

**Authors:** Qiwei Chen, Lan Yang, Qiming Feng, Scott S. Tighe

**Affiliations:** ^1^School of Information Management, Guangxi Medical University, Nanning, Guangxi 530021, China; ^2^School of Humanities and Social Science, Guangxi Medical University, Nanning, Guangxi 530021, China; ^3^Department of Criminal Justice, Western Oregon University, Monmouth, OR 97361, USA

## Abstract

*Background*. Township hospitals in China provide rural communities with basic but much needed critical health care services. The doctors working in these hospitals often feel unsatisfied when considering their work schedules and financial rewards.* Method*. To explore job satisfaction of health workers in a township hospital, a qualitative study was conducted of 39 doctors from five township hospitals in Guangxi Zhuang Autonomous Region. The goal was to understand the level of job satisfaction of doctors and to make recommendations for improvements.* Results*. About 75% (28/39) of the doctors expressed negative attitudes related to their work conditions. Slightly more than half (22/39) mentioned they should receive greater compensation for their work and more than one were seriously considering other options. Many participants (35/39) showed their satisfaction about the achievement of serving as a doctor.* Conclusion*. Their main concerns related to job satisfaction included working conditions, financial rewards, and the doctor's relationships with patients. Increasing the incomes and fringe benefits of healthcare workers, improving their work conditions, and providing training and continuing education opportunities would help rural clinics retain doctors and eliminate the current unsatisfactory conditions. The findings also highlight the need for the government to increase financial support of township hospitals.

## 1. Introduction

Township hospitals serve as essential and important health resource centers in rural areas that lack access to medical care and skilled physicians. Doctors who work in these facilities primarily provide public health services, diagnose common diseases, and treat rural residents in these health centers. With the current level of rapid economic growth and social development, China's health system faces new challenges, such as the increased demands and expenditures for health care, inefficient use of health care resources, and an imbalanced distribution of the medical workforce. The central government of China passed a landmark program related to the health system in 2009, which was aimed at improving both health care for all citizens by strengthening disease control and the primary care system [1, 2]. The new medical reform program calls for building “a strong rural health service network while improving the wages of rural doctors.”

Job satisfaction can be defined as having a positive emotional state resulting from a person's appraisal of his or her job. The media strongly dispute the supposed high level of job satisfaction experienced by medical doctors and the attractiveness of careers in the medical profession for China's younger generation. Some have reported widespread dissatisfaction and low morale among medical doctors in China [3]. Liu et al. (2010) reported on the mean job satisfaction for staff in township health centers in China; they determined a score of 83.3 (range: 0, extremely dissatisfied, to 100, extremely satisfied). Many studies also report that the overall workload and financial rewards for those workers significantly affected the job satisfaction of medical workers [4–7]. However, while a large number of studies have focused on the relationship between job satisfaction and factors that influence that satisfaction, evidence in the context of employees for China's township health centers is limited.

Guangxi Zhuang Autonomous Region (Guangxi) is a relatively underdeveloped autonomous region in South China bordering Vietnam; the region has a chronic shortage of health professionals, especially in rural communities. At the same time, the health services that are available in city level hospitals of Guangxi are not accessible by the rural population because of both financial hardships and the distance involved. Thus, the development and improvement of health care has to rely on primary healthcare providers who are employed in township health centers in rural Guangxi. Guangxi has a total of 53,400 medical personnel who work in over 1,000 township health centers, accounting for 26.5% of all the doctors in the region. The professional health care teams at the township hospital need to ensure the quality and sustainability of the medical services they provided. However, these hospitals in the rural areas are typically understaffed for various reasons including low salary, high workloads, and stress. A growing number of physicians are leaving or intending to leave their organizations in this rural part of China because of inadequate job satisfaction [3]. We conducted a qualitative study with doctors from different township hospitals in a health reform pilot project of Guangxi by focus group discussions (FGDs). The study aims to understand the level of job satisfaction as felt by primary health care providers. We aim to suggest concrete areas for improvements to policy makers, so as to help advance the development of basic health care services in rural China.

## 2. Methods

### 2.1. Sample and Settings

We recruited participants who are doctors and employed in five selected township hospitals in Wuming (a health reform pilot project area) of Guangxi Autonomous Region, China. These included the Matou, Shuangqiao, Xianhu, Yuquan, and Liangjiang township health centers.

### 2.2. Procedures

Participants were recruited during March–April 2016 through the hospital liaisons in each township health center and had previously participated in a protocol development workshop at an earlier stage. The liaison scheduled focus group discussions (FGDs) and was provided with verbal and written background information related to the study and knew the selected characteristics of people we were searching for to participate in the FGDs. Some selection criteria were as follows: doctor employed in a township health center, willing to deliver consent to participate documentation during the FGDs, and was able to communicate in the Mandarin Chinese or the local dialect (Cantonese).

### 2.3. Data Collection

A team of graduate students at the School of Information Management with experience in conducting qualitative research served as interviewers and collected the data. To collect data, two interviewers worked as a team, one conducted the FGD and the other recorded detailed notes. They recorded the sessions with a digital voice recorder with permission from the participants. All FGDs were held at the hospital in a private meeting room and lasted for about 50 minutes. The guide included questions and queries on the following six themes: attitudes towards working conditions; views about workload and financial rewards; willingness to provide health care; attitudes towards job achievement; attitudes towards doctor-patient relationships; and measures taken to improve doctors' job satisfaction. To compensate participants' time, each of them was offered a cash amount of RMB 50 (US $7.27). The research was approved by the Ethics Committee of the Guangxi Medical University.

### 2.4. Analyses

The interviewers discussed and summarized the main content of each FGD and reviewed their notes immediately after each FGD. These debriefings were useful (i) to identify the most crucial themes and ideas and (ii) to evaluate the demand for possible modifications in the subsequent FGDs. The audio recordings were reviewed and transcribed for each group. Two members of the research team coded each transcript independently, with discrepancies resolved through consensus. The process of coding involved identifying central themes and highlights on the transcripts. All additional notes taken during the course of the FGD were examined to identify the diverse themes presented during these qualitative discussions.

## 3. Results

Five FGDs were conducted individually with 39 doctors in five township health centers in Wuming of Guangxi, China. Twenty-three (59%) of the participants were males and sixteen (41%) were females. The level of education varied from those who had a degree from a junior college (77%) to a bachelor's degree and above (23%; Table 1).

The findings revealed six main themes relating to doctors' job satisfaction in township health centers: attitudes towards working conditions; views related to workload and financial rewards; willingness to provide health care; attitudes towards job achievement; attitudes towards doctor-patient relationships; and measures taken to improve doctor's job satisfaction. These themes are described below as supplemented by participants' statements on key themes (Table 2).

### 3.1. Attitudes towards Working Conditions

The participants considered their working conditions to be an important factor related to job satisfaction. According to our study results, quite a large number of doctors (30/39) described a pleasant working environment as a driving factor that would improve their job satisfaction. Meanwhile, the majority of participants (32/39, 89%) complained that the many kinds of medicines and diagnostic devices were squeezed into very a limited space which created a very disorganized situation in the township health centers. One of the doctors said* “I do not have the luxury to rest here during lunch, so after work I have to go home at noon to relax.*”

“*We are too busy at work to keep things neat and orderly, and we do not have enough sanitation workers like the large hospital here because we are experiencing a budget crunch,*” one of doctors who had worked in a township health center for more than 10 years mentioned.

Nine of 39 participants (23%) mentioned that not every doctor has access to air conditioning. “*When you are engaged in work, it is difficult to survive in summer without air conditioning, because it is extremely hot in the summer in Guangxi, with peak temperatures even up to 40*°*C sometimes.*”

Nearly all the respondents (28/39, 72%) expressed negative attitudes towards the entire set of working conditions during the interview; they did emphasize, either directly or indirectly, the importance of improving the working conditions.

### 3.2. Views Related to Workload and Financial Rewards

More than half of participants (22/39) felt the financial rewards were too low and failed to reflect the job's value. “*We are a neglected group among doctors, but we solve the most difficult healthcare problems of rural residents who account for the largest proportion of the population in China. We deserve better financial rewards,*” a participant indicated during the interview.

In terms of work load, the doctors in township health centers work 6.5 days a week on average, with 10.21 hours a day.* “I earned below 2000 RMB (USD 303) per month, and sometimes I work more than 14 hours in one day,” *one doctor said.

A few participants (6/39) pointed out that some medical charges are too low. “*Take the registration fee for example. We only charged patents 5 RMB in total (less than one USD) under the regulations. That is far too low,*” one doctor added.

These findings indicate that doctors believe that their current remuneration does not match the value of their work. Nearly all the respondents explicitly showed their burning desire to increase their income or subsidies. As one of participants said, “*If the government appropriated more money for the township health centers, it would be quite a motivation for doctors to actively participate in health care.”*

### 3.3. Willingness to Provide Health Care

When asked whether they are willing to provide health care in the township health centers, most of participants (29/39, 74%) were still willing to do it. They considered providing health care as their duty and responsibility.

“*I feel obliged to do it. I am glad to see some of the patients be able to go back to a normal life after taking pills. I feel a sense of achievement,*” one of the participants said.

Other participants (6/39, 15.3%) thought they have to provide health care based on the requirements of the upper-level administration. Only one respondent expressed that they felt reluctant to provide health care to local people, by saying, “*I do not want to do it. No one will work without rational rewards. I have to work for too long every day. I am considering leaving the profession for another job such as work in business.*”

### 3.4. Attitudes towards Doctor-Patient Relationships

Doctor-patient relationships serve as an important component of the social exchange relationship for doctors. When it comes to doctor-patient relationships, quite a few of the participants (30/39, 77%) expressed satisfaction with their current relationships. “*Most of the patients here are local farmers. They are honest and full of integrity. They followed our advice and showed their appreciation to us. I felt a sense of recognition,*” a doctor said during the interview.

A few of the participants (7/39, 18%) indicated that the patients could not understand the doctor's work.* “Sometimes they cursed and shouted at us. Even worse, some patients doubted the value of our medical services*,” a female participant said.

A rewarding relationship with patients is very important to doctors. “*An acknowledgement, an appropriate word of appreciation from patients, will increase my job satisfaction. Sometimes, a simple, ‘Thank you, doctor,' really warms my heart after providing medical treatment,*” one doctor added.

### 3.5. Attitudes towards Job Achievement

Most participants (35/39, 91%) expressed satisfaction about the achievement of being a doctor. “*When my patients are cured after treatment, I feel so fulfilled and delighted. One patient still maintains contact with me. Our friendship began when he came to me with appendicitis. He has been well for five years now,*” one of the participants said.

Another female doctor who had worked in a township health center for more than 20 years said, “*The sense of being fulfilled topped my wish list, it did brighten my enthusiasm for working*.”

However, a few participants mentioned they felt less content related to their job achievements. A male doctor said, “*I am only able to provide primary health care to local people, practicing a mixture of basic Western medicine and Traditional Chinese Medicine. However, when it comes to sophisticated conditions, we cannot handle them.*”

### 3.6. Measures Taken to Improve Doctors' Job Satisfaction

Most participants proposed some reasonable measures to brighten the doctor's situation in the township health centers. One of the most strongly supported measures by respondents is to increase the financial support to township hospitals. “*If more money were spent on the infrastructure here, our working conditions could be improved greatly, and I would be more pleased about working here*,” one participant said.

Another participant added, “*I have sacrificed too much for my work. If more work has to be done on weekends, I wish some subsidies could be offered to compensate me for my time and energy.*”

Several participants (16/39, 41%) indicated that more doctors should be recruited to the township health centers. “*We lack the doctors we need to provide adequate services. The shortage has pushed us to work longer. If more doctors could join us, that may ease our burdens,*” a male doctor mentioned.

## 4. Discussion

Job satisfaction has been defined in many ways. “Some believe it is simply how content an individual is with his or her job, in other words whether or not they like the job or individual aspects the job, such as the nature of the work” or the type of supervisor they have [8]. This feeling influences their job performance. Health care providers engaged in the township hospitals are vital to increasing the availability of health services across the rural population. This qualitative research helps us to better understand the attitudes of health workers and their feelings related to job satisfaction when working in township health centers. The results show that quite a large number of doctors expressed pessimistic and negative attitudes towards their working conditions. Having a place for doctors to take a nap at noon and having an appropriate temperature in the working place matters to most of health care providers in the township hospital.

Financial rewards include all of the monetary payments that a doctor receives for doing his/her job. The salary that a doctor receives, as a tangible value, is the most obvious benefit doctors receive for their effort in terms of an economic relationship. As a result, satisfaction with financial rewards is often referred to as “pay satisfaction” [9]. The respondents in our interviews commented that their salaries are relatively low based on their workload and they received no bonuses at all. Early studies have reported that differences in salary can trigger employees' negative or positive emotional reactions, which influences a number of behavioral and attitudinal variables such as their behavior at work, job satisfaction, organizational commitment, and anticipated turnover [10–12]. The government should increase the income of healthcare workers and their fringe benefits along with improving their work site conditions. Apart from that, more incentives should be provided, including providing training and continuing education opportunities and improving the clinics and work space for doctors in order to eliminate unsatisfactory conditions in the workplace.

Extra work and limited funding were mainly obstacles in providing adequate health care. However, most of the doctors were still willing to provide healthcare services in the township health centers because of their feelings of professional responsibility and an achievement sense. A statement issued by the Chinese central government reported that, “The guidance on strengthening the establishment of a village doctor team” required local government to formulate and improve the pension policies for village doctors, in order to enhance the levels of medical practice those local doctors can provide. The health care provider in the township health center plays a significant role in rural areas. Their practice of medicine and full involvement in healthcare enabled the system of cooperative health care to embark on the path of rural development while employing Chinese characteristics such as low-cost with high-quality healthcare [5].

## 5. Conclusion

The findings of this qualitative study among doctors indicate that their satisfaction related to working in health care centers varies from individual to individual. Working conditions, financial rewards and the doctor's relationships with patients are their main concern when it comes to job satisfaction. The findings also highlight the need for the government to increase financial support of township health centers.

The strength of our study was not only in the diverse range of respondents in terms of age, gender, education, and years of experience, but also in the locations used to gather varying views and ideas, in five different township health centers. However, one limitation of the present study was that all participants were recruited from the less developed areas of Guangxi Province, limiting our ability to generalize the finding to potential participants in other developed areas of China. However, this represents the first quantitative study that focuses on the thoughts and attitudes among health care providers in rural China. The findings should encourage more research in this area to promote the job satisfaction of healthcare workers who provide healthcare at health care centers in rural China.

## Figures and Tables

**Table 1:** Demographic characteristic of FGD participants (*n* = 39).

Township hospital	Matou *n* = 7	Shuangqiao *n* = 7	Liangjiang *n* = 8	XianHu *n* = 9	Yuquan *n = 8*
Gender					
Male	4	5	4	5	5
Female	3	2	4	4	3
Mean age	47	39	42	38	45
Years of work	15.0 ± 6.05	10.3 ± 7.22	17.26 ± 5.80	14.6 ± 3.0	19 ± 5.1
Education attainment					
Junior college or below	6	5	6	7	6
Bachelor's degree or above	1	2	2	2	2

**Table 2:** Typical statements made by doctors by key themes.

	Attitudes towards working condition	Views about Workload and financial rewards	Willingness to provide health care	Attitudes towards doctor-patient relationship	Attitudes towards job achievement	Measures taken to improve doctors' job satisfaction
Matou	Countless kinds of medicine and medical devices were very disorganized	My salary is too low to support my family spending, and what's worse, I am always too busy to spend time with my children	I definitely want to provide health care here because I have lived here for over 30 years; many of the patients are my fiends even some of them are my neighbors	Most of the patients here are local farmers; they are honest and full of integrity; they followed our advice and showed their appreciation to us; I felt a sense of recognition	After my patients become well after treatment, I feel so fulfilled and delighted. Some patient still contact me; our friendship began when they came to me	More money spent on the infrastructures here, to improve working conditions

ShuangQiao	Without air conditioning in summer, it was extremely hot in office, and many patients also complained	Many of my colleges are considering leaving for other careers Because of the high work∖load and low financial rewards in township health center	After treating the patients, I feel a sense of achievement; working here can fulfill my professional goals	Sometimes they cursed and shouted at us; what's worse, some patients doubted our medical service	I am just able to provide primary health care to local people, practicing a mixture of basic western medicine and Traditional Chinese Medicine	I sacrificed too much for my work; if the workload increases on weekends, some subsidies could be offered to compensate for my time and energy

Liangjiang	No bed for doctors to rest after an exhausting period of work shift	Sometimes I felt stressed and what I gained cannot be measured in terms of money	I am familiar to the local people; they respect us and working here was so relax and the medical work goes smoothly	“An acknowledgement, an appropriate word of appreciation from patients, would increase my job satisfaction; sometimes, a “thank you, doctor” really warms me after medical treatment”	When it comes to sophisticated conditions, we cannot handle it, we felt so helpless	We lack doctors; shortage has pushed the limited number of doctors to work longer; if more doctors should join us, may be it could ease our burdens

Xianhu	There is not enough sterilized equipment to clean the workplace	Compared with the doctors in city-level hospitals, ours are pretty low	Definitely yes, but more financial subsidies should be offered	Some patients yelled at us and took our efforts for granted	This job brought my full my full potential, I felt fulfilled	—

Yuquan	Enlarge our office would be better for work	According to my workload, my pay should be raised	The local people are very nice, we are pretty close, I am glad to provide what I can	—	I felt fulfilled if my patients got well and got back to work	It would be great if working conditions were improved

## Abbreviations

FGDs: Focus group discussions.

## Data Access

Please contact the corresponding author for data requests.
